# Formulation of Chemically Defined Media and Growth Evaluation of *Ligilactobacillus salivarius* ZJ614 and *Limosilactobacillus reuteri* ZJ625

**DOI:** 10.3389/fmicb.2022.865493

**Published:** 2022-05-06

**Authors:** Iliya Dauda Kwoji, Moses Okpeku, Matthew Adekunle Adeleke, Olayinka Ayobami Aiyegoro

**Affiliations:** ^1^Discipline of Genetics, School of Life Sciences, College of Agriculture, Engineering and Sciences, University of KwaZulu-Natal Westville Campus, Durban, South Africa; ^2^Gastrointestinal Microbiology and Biotechnology Unit, Agricultural Research Council-Animal Production Institute Irene, Pretoria, South Africa; ^3^Unit for Environmental Sciences and Management, North-West University, Potchefstroom, South Africa

**Keywords:** defined medium, minimal nutritional requirement, growth kinetics, probiotics, lactic acid bacteria

## Abstract

Lactic acid bacteria are increasingly becoming important dietary supplements due to their health benefits when consumed in adequate quantity. The increasing attention on these important microbes has necessitated an in-depth understanding of their physiological processes, such as nutritional requirements and growth patterns, to better harness their probiotic potentials. This study was carried out to determine the nutritional requirements for the growth of *L. salivarius* ZJ614 and *L. reuteri* ZJ625 from a chemically defined medium and evaluate growth kinetics by fitting different sigmoidal growth models. The complete CDM contains 49 nutritional ingredients such as glucose, Tween 80^®^, mineral salts, buffers, amino acids, vitamins, and nucleotides at defined concentrations. In addition, the minimal nutritional requirements of the isolates were determined in a series of single-omission experiments (SOEs) to compose the MDM. Growth curve data were generated by culturing in an automated 96-well micro-plate reader at 37°C for 36 h, and photometric readings (optical density: OD_600_) were taken. The data were summarized in tables and charts using Microsoft Excel, while growth evaluation was carried out using open-source software (*Curveball*) on Python. The results revealed that omission of the amino acids, vitamins, and nucleotides groups resulted in 2.0, 20.17, and 60.24% (for *L. salivarius* ZJ614) and 0.95, 42.7, and 70.5% (for *L. reuteri* ZJ625) relative growths, respectively. Elimination of the individual CDM components also indicates varying levels of growth by the strains. The growth curve data revealed LogisticLag2 and Baranyi–Roberts models as the best fits for *L. reuteri* ZJ625 and *L. salivarius* ZJ614, respectively. All the strains showed appreciable growth on the CDM and MDM as observed in de Man–Rogosa–Sharpe (MRS) broth. We also described the growth kinetics of *L. reuteri* ZJ625 and *L. salivarius* ZJ614 in the CDM, and the best models revealed the estimated growth parameters.

## Introduction

Lactic acid bacteria (LAB) are commonly associated with the gastrointestinal tracts (GIT) of humans and animals (Riboulet-Bisson et al., 2012) and can modulate some important physiological functions when consumed as probiotics (VidyaLaxme et al., 2014; Kwoji et al., 2021). Previous studies have shown probiotics to enhance feed digestibility, weight improvement, quality meat and milk yields, low methane release, and better health in animals (Elghandour et al., 2015; Deng et al., 2018; Mani et al., 2021). Some microbes with probiotic potentials include strains from the genus *Bifidobacterium, Bacillus, Enterococcus, Micrococcus, Pediococcus, Streptococcus, Lactobacillus, Propionibacterium, Saccharomyces, Debaryomyces*, and *Photobacterium*, among others (De et al., 2014; Babot et al., 2018). Species and strains from the *Lactobacilli* genus are among the most widely known and used probiotics (Li et al., 2020). Different *Limosilactobacillus reuteri* (formerly: *Lactobacillus reuteri*) strains are effective as probiotics with no potential of transferring antibiotic resistance factors (Rosander et al., 2008). Through their mechanism of anti-inflammatory actions, *L. reuteri* plays a vital role in alleviating gastroenteritis in children (Dinleyici et al., 2014; Szajewska et al., 2014; Xu et al., 2015) and infantile colic, especially at 2–3 weeks of age (Savino et al., 2010; Szajewska et al., 2014; Chau et al., 2015; Mi et al., 2015; Xu et al., 2015). *Ligilactobacillus salivarius* (formerly *Lactobacillus salivarius*) is an important probiotic bacterium commonly isolated from humans', pigs', and birds' GITs and known to produce bacteriocins (Zhang et al., 2011). Some *L. salivarius* strains improve the hosts' immunity by stimulating interleukin (IL)-10, immunoglobulins levels, and increased production of natural killer cells (monocytes) (Sierra et al., 2010). *L. salivarius* also stimulates an increase in the level of immunocompetent cells and enhance the expression of genes associated with IL-6 in porcine's intestines (Zhang et al., 2011).

Lactic acid bacteria have complex nutritional requirements and require several nutrients, including carbohydrates as energy and carbon sources, mineral salts, vitamins, amino acids, and nucleotides for their growth (Hébert et al., 2004b; Wegkamp et al., 2010). Naturally, LAB satisfy their extensive nutritional requirements from complex culture media [e.g., de Man–Rogosa–Sharpe (MRS) medium] by utilizing non-specific compounds available in peptone, meat, and yeast extracts (De Man et al., 1960; Hébert et al., 2004b). However, enriched media are not suitable for determining nutritional requirements because they contain undefined components, making results interpretation difficult in analytical studies (Kim et al., 2012; Aller et al., 2014). Complex nutrients also make it hard to differentiate media components (yeast, peptone, or beef extracts proteins) from actual expressed proteomes or metabolites. Although enriched and semi-defined media often yield high population density, using these media types in physiological works focusing on metabolism analyses makes data interpretation a hard task to perform (Cocaign-Bousquet et al., 1995; Zhang et al., 2009). Therefore, defined media that support growth are useful in designing reproducible biochemical, genetic, and analytical studies (Hébert et al., 2004b; Zhang et al., 2009). The systematic addition or omission of CDM components enables specific nutritional and regulatory requirements for growth and targeted biochemical pathways. Furthermore, complex interactions among media components are minimized, making the culture environment more reproducible (Van Niel and Hahn-Hägerdal, 1999), and could enable an understanding of microbial growth kinetics.

Mathematical models are important in predictive microbiology for studying microbial growth kinetics based on their responses to environmental perturbations (Pla et al., 2015). For effective applications of predictive microbiology, mathematical models that sufficiently explain bacterial behaviors are crucial. Different equations and sigmoidal models are useful as growth functions and vary in their simplicity and the number of the equation's parameters (Pla et al., 2015). Previous studies have related the nature of several growth models from different perspectives, including the mathematical measures of goodness of fit (Zwietering et al., 1990; Buchanan et al., 1997; Baty and Delignette-Muller, 2004). However, several reports have shown that Gompertz, Logistic, Baranyi, Richards, and three-phase linear models are the most used models for microbial growth evaluation (López et al., 2004; Jewell, 2012; Huang, 2013). Bacterial growth curves normally explain the populations of cells in broth culture per time and are generated through quantifying optical density (OD). The Malthusian parameter (growth rate) in the logarithmic (exponential) growth phase is determined using the gradient of the log of the growth curve and is the easiest way to infer fitness (Ram et al., 2019). However, the exponential growth rates do not wholistically express the dynamics of a typical growth curve (Hall et al., 2014), hence making growth rates poor predictors of relative fitness since they are not uniform throughout the entire growth.

Therefore, this study employed a computational technique to estimate growth parameters from growth dynamics to predict growth in a mixed culture by fitting estimated parameters from monocultures data. This work also evaluates the nutritional requirements of *L. salivarius* ZJ614 and *L. reuteri* ZJ625 to design a CDM and MDM as a pre-stroll for in-depth “omics” studies of the isolates. Furthermore, we study the growth kinetics of the isolates on the designed chemically defined medium using different mathematical non-linear models.

## Materials and Methods

### Inoculum Preparations

The isolates were revived by cultivating in fresh MRS broth (Oxoid™, Thermo Scientific™, Hampshire, UK) prepared in an anaerobic system (Forma Scientific Anaerobic System, model 1024, Thermo Scientific). The inoculum was prepared after determining the colony-forming unit (CFU) and dry-weight mass per mil. The final working volume was determined by standardizing the growth of each isolate using 0.5 McFarland's standard (approximately containing 1.5 × 10^8^ CFU) for subsequent use as the inoculation volume in the CDM and minimal essential nutrient determination experiments.

### Formulation of CDM and MDM for the Culturing of *L. salivarius* ZJ614 and *L. reuteri* ZJ625

A total of 49 (Table 1) nutrients previously used to develop defined media based on the genomic metabolic pathways for other LAB strains were adopted in these studies (Chervaux et al., 2000; Hébert et al., 2004a; Zhang et al., 2009; Aller et al., 2014; Castañeda-Ovando et al., 2019). All the chemicals used were analytical grades (purchased from Glentham Life Sciences LTD, Corsham, UK). Most components were prepared as concentrated stock solutions and stored at 4°C except FeCl_3_, cysteine, and tryptophan, which were freshly prepared before each use. The stock solutions were either sterilized using an autoclave (heat-stable substances) or filter-sterilized (heat-labile substances) using a 0.22-μm syringe-driven filter (Millex^®^, 33mm with Durapore^®^ Membrane, Merck Millipore Ltd., Ireland) (Supplementary Table S1). The water-insoluble amino acids, vitamins, and nucleotides were dissolved using acidic (HCl and H_2_SO_4_) or alkaline (NaOH) solutions. All the stock solutions were prepared using distilled water unless otherwise stated. The acid and alkaline solutions were also used for adjusting the pH of the different composed media.

**Table 1:** Composition of chemically defined and minimally defined media developed for *L. salivarius* ZJ614 and *L. reuteri* ZJ625.

**Components**	**Concentration (g/L)**			
	**CDM**	**MDMLS**	**MDMLR**	**MDMLSLR**
Glucose	15	15	15	15
Tween 80^®^	1	1	1	1
K_2_HPO_4_	3	3	3	3
KH_2_PO_4_	3	3	3	3
MgSO_4_.7H_2_O	2.5	2.5	2.5	2.5
MnSO_4_.4H_2_O	0.025	0.025	0.025	0.025
CoCl_2_.6H_2_O	0.001	–	–	–
CaCl_2_.2H_2_O	0.1	–	–	–
ZnSO_4_.7H_2_O	0.01	–	–	–
H_3_BO_3_	0.001	–	–	–
KCl	0.5	–	–	–
NH_4_Cl_2_	1	1	1	1
Sodium acetate	5	5	5	5
CuSO_4_.5H_2_O	0.001			
FeSO_4_.7H_2_O	0.02	0.02	0.02	0.02
**Amino acids**		
L-Alanine	0.1	–	0.1	0.1
L-Isoleucine	0.2	0.2	0.2	0.2
DL-Methionine	0.1	0.1	0.1	0.1
L-Valine	0.1	0.1	–	0.1
L-Arginine	0.1	0.1	0.1	0.1
L-Phenylalanine	0.1	0.1	0.1	0.1
L-Histidine	0.25	0.25	0.25	0.25
L-Proline	0.1	–	–	–
Glycine	0.1	0.1	0.1	0.1
L-Lysine	0.1	–	–	–
L-Threonine	0.1	0.1	0.1	0.1
L-Leucine	0.1	0.1	0.1	0.1
L-Glutamine	0.2	0.2	0.2	0.2
L-Glutamic acid	0.2	0.2	0.2	0.2
L-Asparagine	0.2	0.2	0.2	0.2
L-Aspartic acid	0.2	–	–	–
L-Tyrosine	0.1	0.1	0.1	0.1
L-Serine	0.1	0.1	0.1	0.1
L-Tryptophan	0.1	0.1	0.1	0.1
L-Cysteine	0.2	0.2	0.2	0.2
**Nucleic acid bases**		
Guanine	0.01	–	0.01	0.01
Adenine	0.01	–	–	–
Xanthine	0.01	0.01	–	0.01
Thymidine	0.01	–	0.01	0.01
Uracil	0.01	0.01	0.01	0.01
**Vitamins**		
Nicotinic acid	0.001	–	–	–
Thiamine HCl	0.001	–	–	–
Cyanocobalamin	0.001	0.001	0.001	0.001
Pyridoxal	0.002	0.002	0.002	0.002
Pantothenic acid	0.001	0.001	0.001	0.001
Riboflavin	0.001	0.001	0.001	0.001
Folic acid	0.001	–	–	–
P-Aminobenzoic acid	0.01	–	–	–
Biotin	0.01	–	–	–

The CDM was prepared by adding the solutions in the following sequence: distilled water, followed by phosphate buffer, sodium acetate, ammonium chloride, vitamins, amino acids, nucleotides, Tween 80^®^, Magnesium sulfate, iron chloride, manganese sulfate, and glucose, as earlier described (Hébert et al., 2004a; Zhang et al., 2009). The pH was adjusted to 6.14 to match the freshly prepared MRS broth using 1N HCl and 10N NaOH. Finally, the medium was filter-sterilized using a 0.22-μm syringe-driven nylon filter and used within 24 h. Thirty-seven (37) single-omission experiments (SOEs) were performed to determine the minimal essential nutritional requirements of *L. reuteri* ZJ625 and *L. salivarius* ZJ614. As an initial stage of the SOEs, the amino acids, vitamins, and nucleotides groups were omitted to evaluate the necessity of each group. Subsequently, individual amino acid, vitamin, or nucleotide was omitted from the medium to determine their necessity for the growth of the strains. If a particular nutrient was found to be essential or stimulatory for the growth of the isolates, it is reincorporated back into the medium. A nutrient is essential if omission results in growth <40% of that obtained in the complete CDM, stimulatory (between 40 and 80%), and non-essential if >80% of CDM growth. In each case, the SOE was repeated to confirm the necessity of each medium component.

### Growth Curve Experiment of *L. reuteri* ZJ625 and *L. salivarius* ZJ614 in the CDM

The growth curve experiment was performed by inoculating ~1.5 × 10^8^ CFU/mL of 18-h culture of the bacterial cells after adjusting to 0.5 MacFarland's standard. Bacterial cells were washed twice using 0.85% (w/v) sterile saline solution after centrifuging (12,000 × g, 5 min) to rid MRS medium components before inoculating the defined media. The cultures were incubated at 37°C for 24 h in triplicates alongside negative control (uninoculated CDM) and MRS plus the isolates as the positive control. The growth curve experiment was performed by culturing in a 96-well microtiter plate by turbidometry at 600 nm using the Multiskan-Go^®^ (Thermo Fisher Scientific, Vantaa, Finland) spectrophotometer at 37°C for 36 h. A total of 144 OD readings were taken at 15-min intervals with continuous shaking. Each isolate was cultured with 40 replicates, and the experiment was performed thrice for repeatability and methodology validation.

### Data Analysis

The data generated were exported and analyzed in Microsoft Excel using the data analysis add-in. The growth curve experiment data were analyzed using *Curveball*, following the method described by Ram et al. (2019). Curveball (http://curveball.yoavram.com) is open-source software coded in Python (Van Rossum and Drake Jr, 1995) containing programmatic and command-line interfaces. The source code employs the use of various Python packages, including *NumPy* (Van Der Walt et al., 2011), SciPy (Jones et al., 2001), Matplotlib (Hunter, 2007), Pandas (McKinney, 2010), Seaborn (Ram et al., 2019), LMFIT (Newville et al., 2016), Scikit-learn (Pedregosa et al., 2011), and SymPy (Meurer et al., 2017).

### Growth Model Fitting

The least squares of the sigmoidal curve fitting procedure in *SciPy* were employed to fit growth models' equations to the growth density data. The Bayesian information criteria (BICs) of various nested models were calculated by attaching the values of the individual growth parameters (Jones et al., 2001; Ram et al., 2019). The BIC was estimated using the equation:


$$\begin{array}{l} BIC=n.\log\left(\frac{\overset{n}{\underset{i=1}{\sum }}\left(N\left(t_{i}\right)-Ñ{\left(t_{i}\right)}^{2}\right)}{n}\right)+k.\log n \end{array}$$

where *k* is the number of models' parameters, *n* is the number of points in the data, *t*_*i*_ is the time points, *N(t*_*i*_*)* is the OD at time *t*_*i*_, and Ñ*(t*_*i*_*)* is the expected population density at time point *t*_*i*_ fitting to the model. The fitted growth model determined the lag phase duration, maximum specific growth rate, and minimum doubling time [*N(t)*] Baranyi-Roberts equation in Table 2. Lag phase duration is the time at which the line tangent to *N(t)* at the point of maximum derivative (i.e., the inflexion point) traverses with *N*_0_, the initial population size (Baranyi, 2010). The maximum specific (i.e., per-capita) growth rate is given by max $\left[\left(\frac{1}{N}\right)\;.\;\left(\frac{dN}{dt}\right)\right]$. The specific growth rate is useful as a metric for comparing different strains or treatments as it is independent of population density. At the same time, the minimum doubling time is the lowest time needed for the microbial population to double, denoted as *N(t)*. The different models employed in this work with their respective equations and estimated parameters are outlined in Table 2.

**Table 2:** Growth models, equations, and estimated parameters.

**Models**	**Equations**	**Free parameters**	**Fixed parameters**	**References**
Logistic	$\frac{dN}{dt}=rN\left(1-\frac{N}{K}\right)\Rightarrow \;$ $N\left(t\right)=\;\frac{K}{1-\;\left(1-\frac{K}{N_{0}}\right)e^{-rt}}$	*N*_0_, *r, K*	*v* = 1 *q*_0_, *m* → ∞	Gilpin and Ayala (1973)
Richards	$\frac{dN}{dt}=rN\left(1-{\left(\frac{N}{K}\right)}^{v}\right)\Rightarrow \;$ $N\left(t\right)=\;\frac{K}{{\left[1-\;\left(1-{\left(\frac{K}{N_{0}}\right)}^{v}\right)e^{-rvt}\right]}^{\frac{1}{v}}}\;$	*N_0_, r, K, v*	*q*_0_, *m* → ∞	Dilao and Domingos (2000)
Baranyi–Roberts	$\frac{dN}{dt}=r\alpha \left(t\right)N\left(1-\;\frac{N}{K}\right)\Rightarrow$ $N\left(t\right)=\;\frac{K}{{\left[1-\;\left(1-{\left(\frac{K}{N_{0}}\right)}^{v}\right)e^{-rvA\left(t\right)}\right]}^{\frac{1}{v}}}\;$ The Baranyi–Roberts model introduced an adjustment function α(*t* ) $\alpha \left(t\right)=\;\frac{q_{0}}{q_{0\;}+\;e^{-m.t}}$ $A\left(t\right):=\overset{t}{\underset{0}{\int }}\alpha \left(s\right)ds$ $=\overset{t}{\underset{0}{\int }}\frac{q_{0}}{q_{0}+\;e^{-vs}}ds$ $=t+\;\frac{1}{v}\log\left(\frac{e^{-m.t}+\;q_{0}}{1+\;q_{0}}\right)$ The above equation was integrated to obtain the following Baranyi–Roberts equation which was used or the growth model fitting: $N\left(t\right)=\frac{K}{{\left[1-\left(1-\;{\left(\frac{K}{N_{0}}\right)}^{v}\right)e^{-r.v.t}\right]}^{\frac{1}{v}}}\;$ Where $A\left(t\right)=\overset{t}{\underset{0}{\int }}\alpha \left(w\right)dw=t+\frac{1}{m}\log\left(\frac{e^{-m.t}+\;q_{0}}{1+\;q_{0}}\right)\;$	*N*_0_, *r, K, v, q*_0_, *m*	*v* = 1	Baranyi and Roberts (1994)

The data from the monocultures were used to simulate a competition model using the following two-strain Lotka–Volterra competition model (Otto and Day, 2007) based on nutrient consumption (Ram et al., 2019) (Table 2):


$$\left\{ \begin{array}{l} \frac{dN_{1}}{dt}=r_{1}\frac{q_{0,1}}{q_{0,1}+e^{-m_{1}t}}N_{1}\left(1-\frac{N_{1}^{v_{1}}}{K_{1}^{v_{1}}}-c_{2}.\frac{N_{2}^{v_{2}}}{K_{1}^{v_{1}}}\right)\\ \frac{dN_{2}}{dt}=r_{2}\frac{q_{0,2}}{q_{0,2}+e^{-m_{2}t}}N_{1}\left(1-c_{1}.\frac{N_{1}^{v_{1}}}{K_{2}^{v_{2}}}-\frac{N_{2}^{v_{2}}}{K_{2}^{v_{2}}}\right) \end{array}\right.$$

where *N*_*i*_ is the density of strain *i* = *1, 2* and *r*_*i*_*, K*_*i*_*, v*_*i*_*, q*_0_, _*I*_, and *m*_*i*_ are the values of the respective parameters for strain *i*, acquired from the growth model fitting (B–R equations) to the monoculture growth curve data. Also, *c*_*i*_ is the competition coefficient, that is, the ratios between interstrain and intrastrain competitive effects. The competition model was fitted to growth curve data from the OD of the individual strains. Since the OD for the mixed culture was unavailable, the competition coefficient *c*_*i*_ was set to 1. The best model fit for each isolate was used to predict a competition curve for the isolates (**Figure 5**). The best model fit selection was based on the Bayesian information criteria (BICs). The BIC was used as a criterion for best-fit selection because of its simplicity and flexibility (Ward, 2008; Ram et al., 2019).

## Results

### Determination of Medium Component Necessities for the Growth of *L. reuteri* ZJ625 and *L. salivarius* ZJ614

The individual components were incorporated to formulate a CDM with the 49 constituents as the first stage to meet the nutritional requirements of *L. salivarius* ZJ614 and *L. reuteri* ZJ625. Second, we determined the necessities of the nutrient groups by omitting each group from the complete CDM. Omission of the amino acids, vitamins, and nucleotides groups resulted in 2.0%, 20.17%, and 60.24% (for *L. salivarius* ZJ614) and 0.95, 42.7, and 70.5% (*L. reuteri* ZJ625) relative growths, respectively (Table 3). The necessity of each medium component was evaluated in a single-omission experiment (SOE) [also known as a leave-one-out experiment (LOO)]. This technique was employed to determine each nutritional factor's necessity to arrive at a minimal defined medium that supports the growth of the strains. A component is considered essential if its omission results in <40% growth than that obtained in the complete CDM. Similarly, a component is stimulatory or non-essential if the obtained growth following its omission is between 40 and 80% or >80%, respectively (Table 3).

**Table 3:** Single omission experiment for the determination of minimum growth requirement by *L. salivarius* ZJ614 and *L. reuteri* ZJ625.

**S. No**.	**Omitted nutrients**	***L. salivarius*** **ZJ614**				***L. reuteri*** **ZJ625**			
		**OD**_**600**_				**OD**_**600**_			
		**0 h**	**24 h**	**Relative growth (%)**		**0 h**	**24 h**	**Relative growth (%)**	
1	Amino acids (AA)	0.071	0.084	2.0	E	0.066	0.045	0.95	E
2	Vitamins (Vit)	0.097	0.837	20.17	E	0.050	2.034	42.7	S
3	Nucleotide bases (NB)	0.098	2.50	60.24	S	0.055	3.35	70.5	S
4	L-Alanine (Ala)	0.099	4.86	110.20	N	0.073	0.475	9.58	E
5	L-Isoleucine (Ile)	0.098	0.421	9.55	E	0.056	0.517	10.42	E
6	L-Arginine (Arg)	0.095	0.16	3.63	E	0.07	0.106	2.14	E
7	DL-Methionine (Met)	0.091	1.56	35.37	E	0.076	1.93	38.91	E
8	L-Valine (Val)	0.083	1.48	33.56	E	0.063	5.22	105.24	N
9	L-Glutamine (Gln)	0.081	0.083	1.88	E	0.069	0.139	2.80	E
10	L-Asparagine (Asn)	0.093	0.072	1.63	E	0.047	0.129	2.60	E
11	L-Leucine (Leu)	0.117	0.144	3.27	E	0.042	0.181	3.65	E
12	L-Glutamic acid (Glu)	0.072	0.114	2.59	E	0.061	0.066	1.33	E
13	L-Lysine (Lys)	0.07	4.12	93.42	N	0.086	4.69	94.56	N
14	L-Threonine (Thr)	0.06	0.046	1.04	E	0.053	0.063	1.27	E
15	L-Phenylalanine (Phe)	0.063	0.264	5.99	E	0.058	3.06	61.69	S
16	Glycine (Gly)	0.08	2.775	62.93	S	0.065	0.882	17.78	E
17	L-Proline (Pro)	0.072	4.14	93.88	N	0.041	4.51	90.93	N
18	L-Histidine (His)	0.049	0.685	15.53	E	0.078	0.61	12.30	E
19	L-Tyrosine (Tyr)	0.106	0.118	2.68	E	0.051	2.11	42.54	S
20	L-Serine (Ser)	0.065	0.82	18.59	E	0.043	0.6	12.10	E
21	L-Cysteine (Cys)	0.065	4.76	107.94	N	0.071	5.27	106.25	N
22	L-Aspartic acid (Asp)	0.105	4.78	108.39	N	0.04	4.73	95.36	N
23	L-Tryptophan (Trp)	0.101	0.581	13.17	E	0.043	0.732	14.76	E
24	Pantothenic acid	0.042	2.33	52.83	S	0.05	1.06	21.37	E
25	Biotin	0.095	4.13	93.65	N	0.063	5.13	103.43	N
26	Cyanocobalamin	0.082	2.78	63.04	S	0.076	3.61	72.78	S
27	Folic acid	0.089	3.66	82.99	N	0.067	5.6	112.90	N
28	Pyridoxal	0.091	2.5	56.69	S	0.059	1.73	34.88	E
29	Nicotinic acid	0.121	4.81	109.07	N	0.06	5.29	106.65	N
30	PABA	0.125	3.61	81.86	N	0.046	5.57	112.30	N
31	Thiamine HCl	0.078	4.09	92.74	N	0.104	4.75	95.77	N
32	Riboflavin	0.093	0.687	15.58	E	0.041	3.54	71.37	S
33	Guanine (G)	0.095	4.45	100.91	N	0.064	3.05	61.49	S
34	Adenine (A)	0.089	3.66	82.99	N	0.066	5.91	119.15	N
35	Uracil (U)	0.063	0.955	21.66	E	0.101	0.131	2.64	E
36	Thiamine (T)	0.098	3.72	84.35	N	0.06	3.94	79.44	S
37	Xanthine (X)	0.079	3.5	79.37	S	0.069	5.16	104.03	N

*E, Essential; S, Stimulatory; N, Non-essential*.

### Determination of Amino Acid Requirements of *L. salivarius* ZJ614 and *L. reuteri* ZJ625

**Figure 1:**
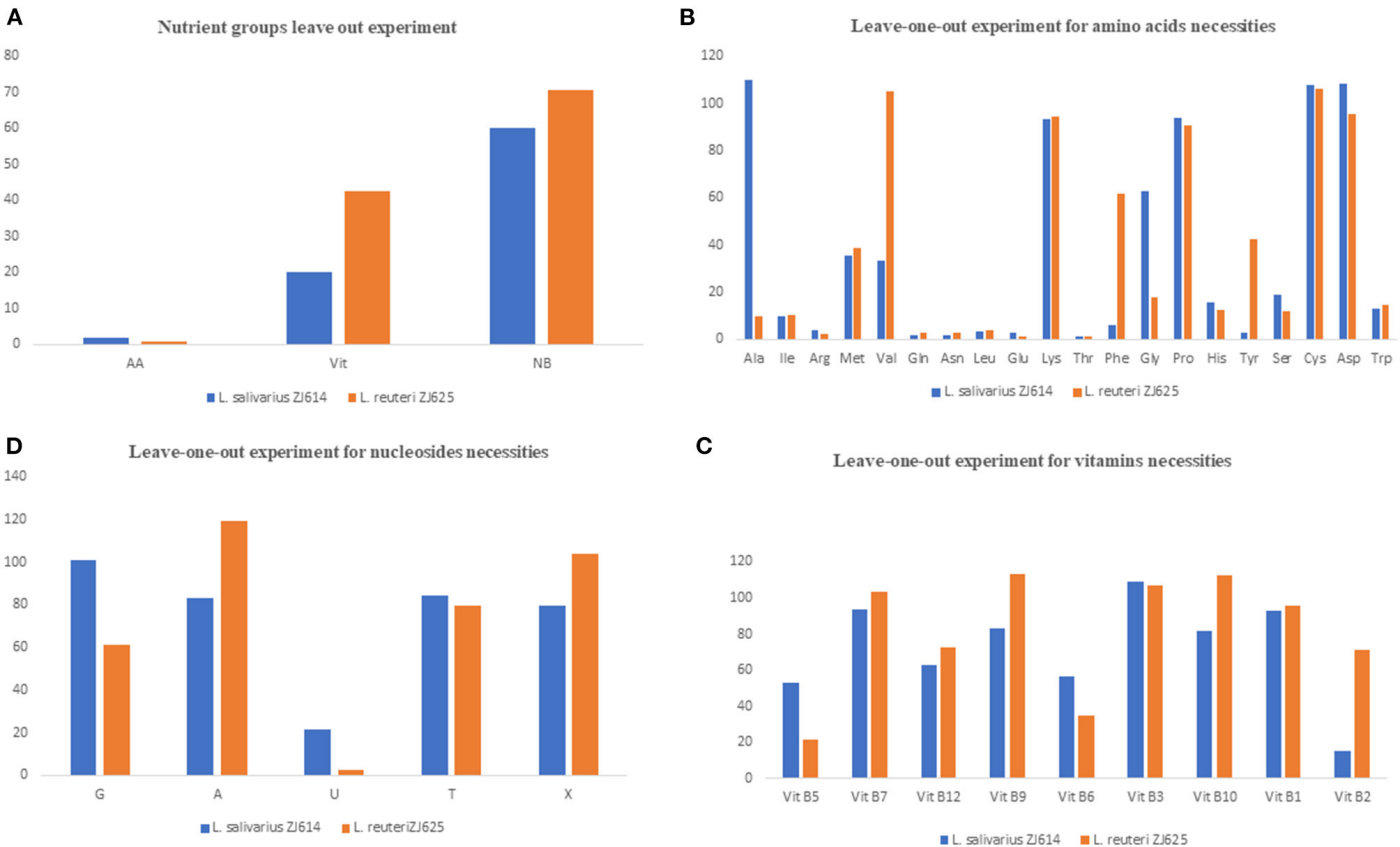
Results of the nutrient group leave out experiments **(A)** and single-omission experiments **(B–D)**. In **(A)**, the complete omission of amino acids results in growth inhibition of <10%, indicating that amino acids are essential for the growth of the isolates. The omission of the nucleotides and vitamins indicates that these two nutrients' groups are stimulatory for the growth of the isolates. **(B)** Is the SOE for the determination of essential amino acids for the growth of the isolates. Omission of most amino acids either results in growth inhibition (essential) or slow and low growth (stimulatory), with only a few such as lysine, proline, asparagine, and cysteine to be non-essential for the growth of both isolates. **(C)** Shows that most of the vitamins are stimulatory for the growth of the isolate as omission results only in low growth. Similarly, the nucleotides are mostly stimulatory for the growth of the isolates.

The results of amino acid omission (Table 3) showed a range of growths by the strains when different amino acids were omitted in a single-omission experiment. Complete omission of amino acids revealed 2.0 and 0.95% relative growths of *L. salivarius* ZJ614 and *L. reuteri* ZJ625, respectively, indicating that the isolates are strict auxotroph for some amino acids (Figure 1A). The omission of the individual amino acids (Figure 1B) showed isoleucine, arginine, methionine, glutamine, asparagine, leucine, glutamine, threonine, histidine, serine, and tryptophan as essential for the growth of both *L. salivarius* ZJ625 and *L. reuteri* ZJ614. However, valine, phenylalanine, and tyrosine are essential for *L. salivarius* ZJ625, unlike *L. reuteri* ZJ614. Similarly, alanine and glycine are essential for the growth of *L. reuteri* ZJ614, unlike *L. salivarius* ZJ625. The other amino acids included are either stimulatory or non-essential for the growth of the strains (Figure 1B).

### Evaluation of Vitamin Requirements of *L. salivarius* ZJ614 and *L. reuteri* ZJ625

The consequence of the omission of the entire vitamins on the growth of *L. salivarius* ZJ625 and *L. reuteri* ZJ614 was evaluated. The results also showed that some vitamins are essential or stimulatory for the growth of *L. salivarius* ZJ614 and *L. reuteri* ZJ625 (Figure 1A). However, *L. reuteri* ZJ614 showed a comparably higher relative growth (42.7%) than *L. salivarius* ZJ625 (20.17%) in the MDM devoid of vitamins, thus indicating that most of the vitamins are stimulatory for the growth of isolates. Subsequently, the isolates were grown in nine different MDMs with one vitamin omitted at a time to determine the effect of each on the growth of *L. salivarius* ZJ625 and *L. reuteri* ZJ614 (Figure 1C). The results of the SOE showed a varying degree of necessity of each of the vitamins, with pantothenic acid and pyridoxal being essential for the growth of *L. reuteri* ZJ614. At the same time, only riboflavin is essential for the growth of *L. salivarius* ZJ625. The other vitamins are either stimulatory or not essential for the growth of the isolates (Table 3).

### Determination of Nucleotide Requirements of *L. salivarius* ZJ614 and *L. reuteri* ZJ625

Experimental omission of the nucleotides group resulted in 60.24% and 70.5% relative growths of *L. salivarius* ZJ625 and *L. reuteri* ZJ614, respectively, thus indicating that the nucleotides group is stimulatory for the growth of the isolates. The SOE showed auxotrophy of the two isolates to uracil. The other nucleotides are either stimulatory or non-essential for the growth of the isolates in SOE (Figure 1C).

### Growth Kinetics of *L. salivarius* ZJ614 and *L. reuteri* ZJ625 on CDM and Models Fitting

**Figure 2:**
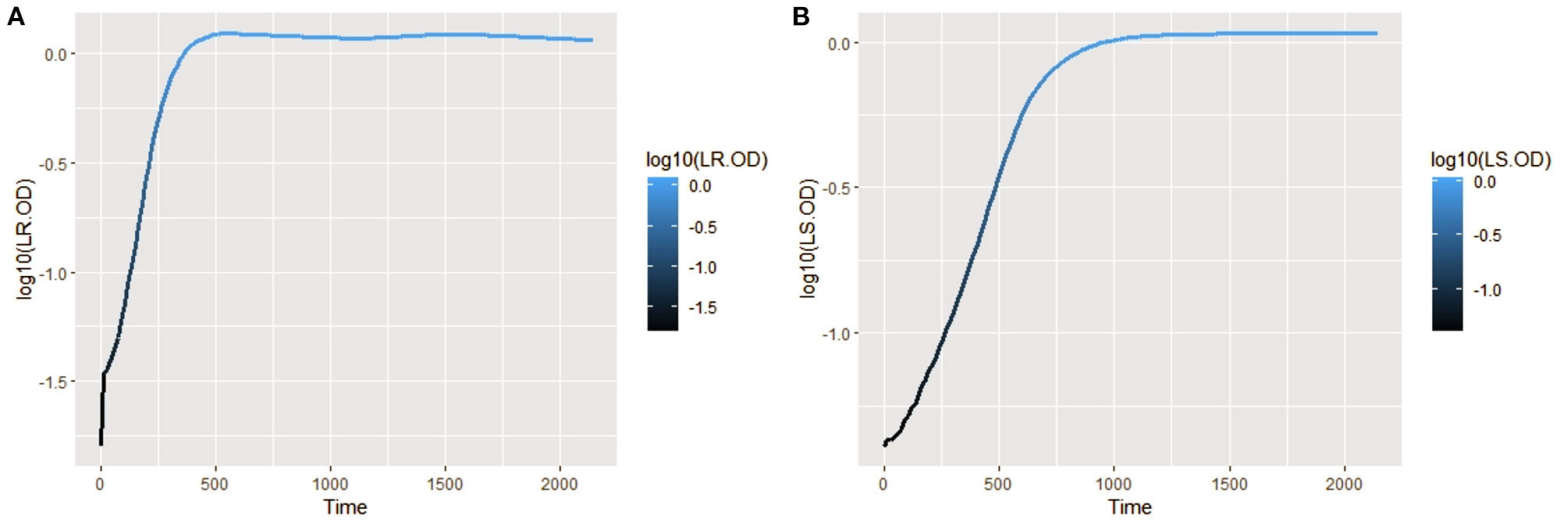
Plots of the log10 of the average growth (OD_600_) of *L. reuteri* ZJ625 **(A)** and *L. salivarius* ZJ614 **(B)** on which the growth parameters (textitN_0_, r, K, v, q_0_, and m) were estimated for fitting on growth model analysis.

The isolates were cultured in CDM (as earlier explained in methodology), and the logarithm of the isolates' growth (OD_600_) was plotted against time in *R* (Hadley, 2016). From the plots, it was observed that *L. salivarius* ZJ614 has a prolonged lag phase which slowly progresses from 0 to 45 min; after that, it increases gradually until about 2 h post-incubation. The growth sharply enters a log phase that lasts for 20 h, marking the end of the log phase and a gradual setting in of stationary phase at about 18–20 h post-incubation (Figure 2A). However, *L. reuteri* ZJ625 displayed a shorter lag phase of between 15 and 30 min of incubation followed by a steep progression into the log phase, 45 min post-incubation. The log phase for *L. reuteri* ZJ625 lasts for about 9 h post-incubation, with the isolate progressing into a stationary phase from 10 h and 15 min post-incubation (Figure 2B).

**Figure 3:**
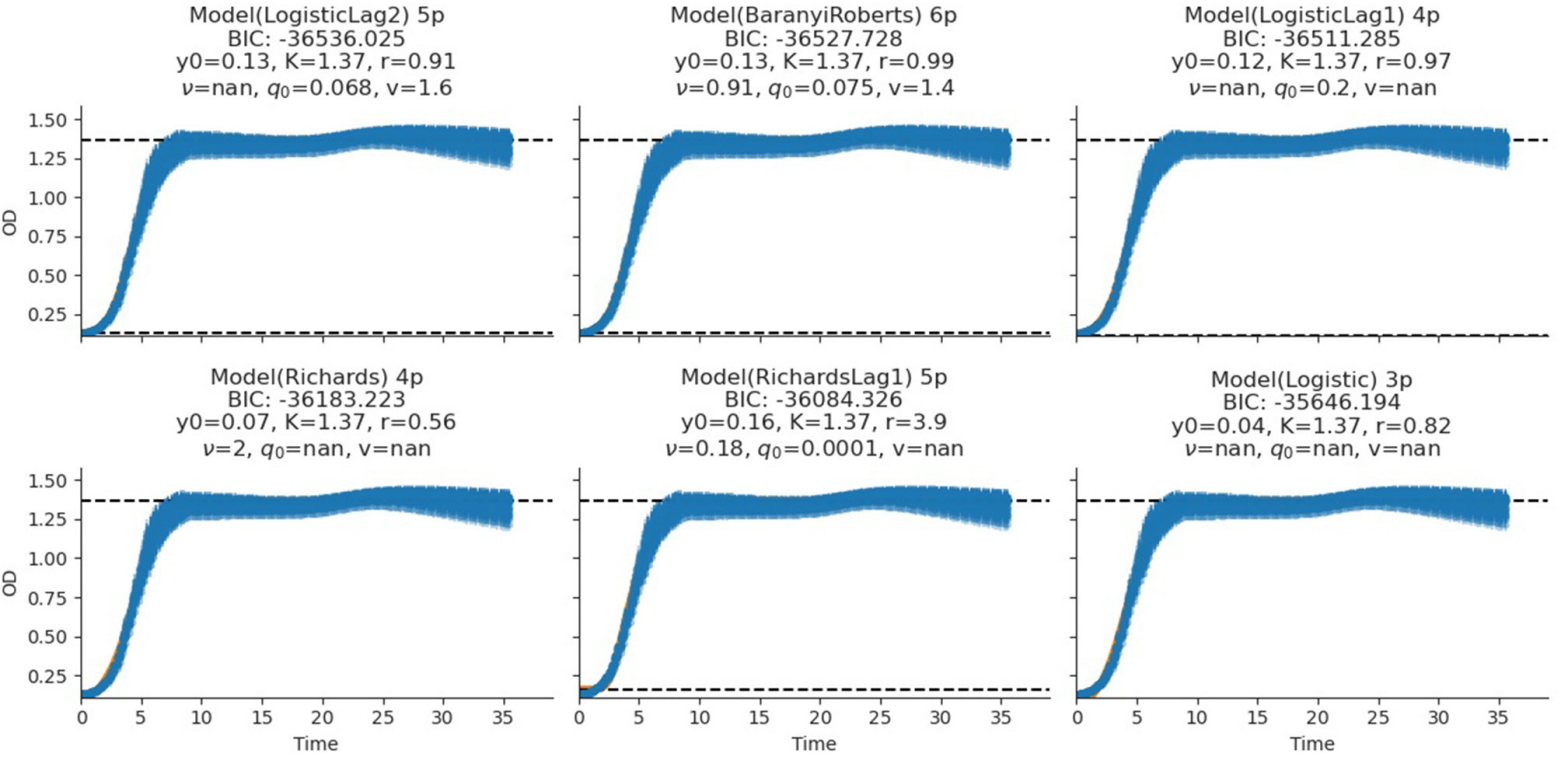
Growth model fits of *L. reuteri* ZJ625. The figures show the estimated growth parameters for the selection of the best model fit. The different growth parameters: initial population (*N*_0_), specific growth rate (*r*), maximum cell density (*K*), deceleration parameter (*v*), initial adjustment (*q*_0_), and the Bayesian information criterion (BIC), were estimated to determine the best model fits.

**Figure 4:**
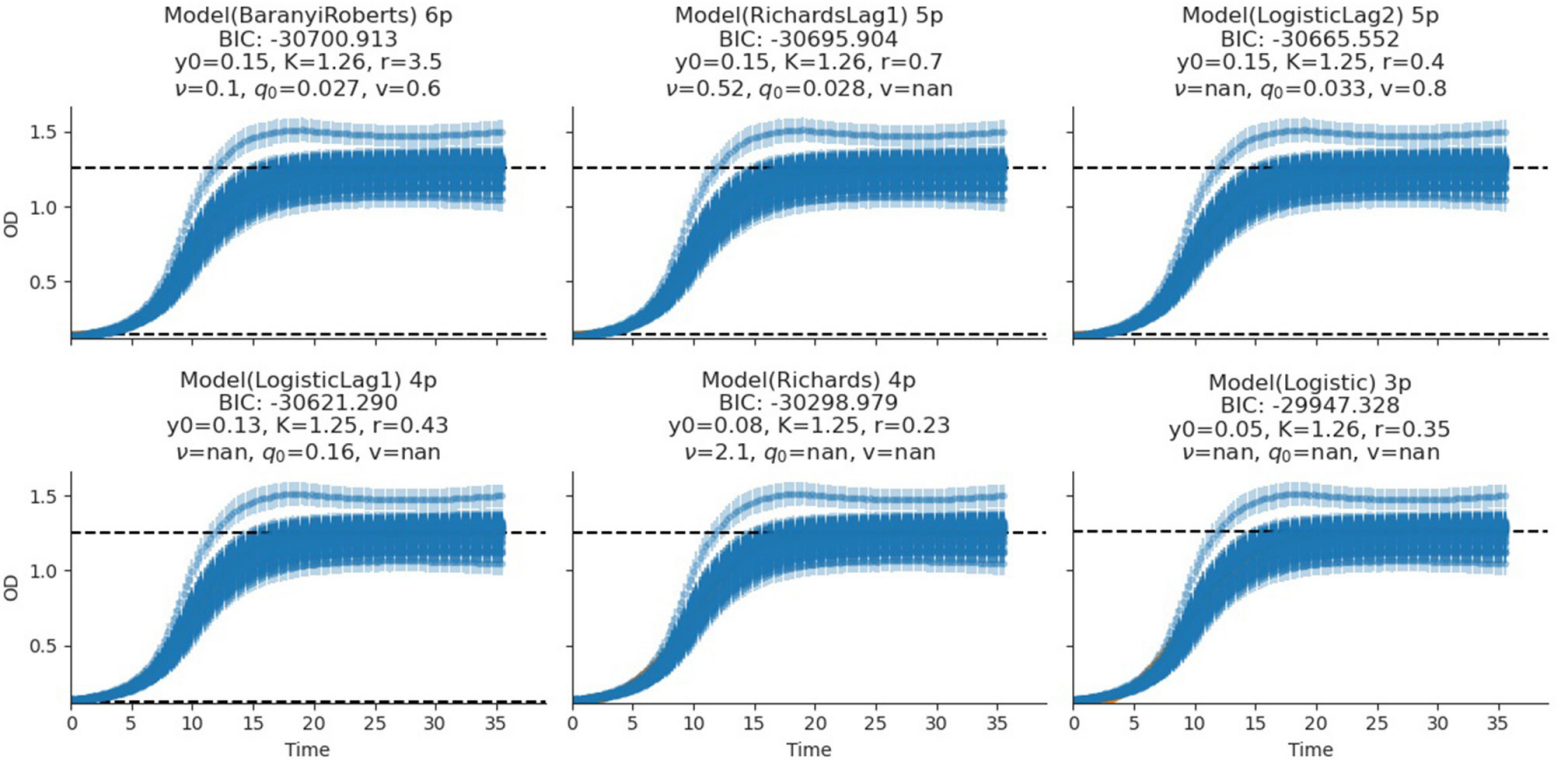
Growth model fits of *L. salivarius* ZJ614. The figures show the estimated growth parameters for the selection of the best model fit. The different growth parameters: initial population (*N*_0_), specific growth rate (*r*), maximum cell density (*K*), deceleration parameter (*v*), initial adjustment (*q*_0_), and the Bayesian information criterion (BIC), were estimated to determine the best model fits.

**Figure 5:**
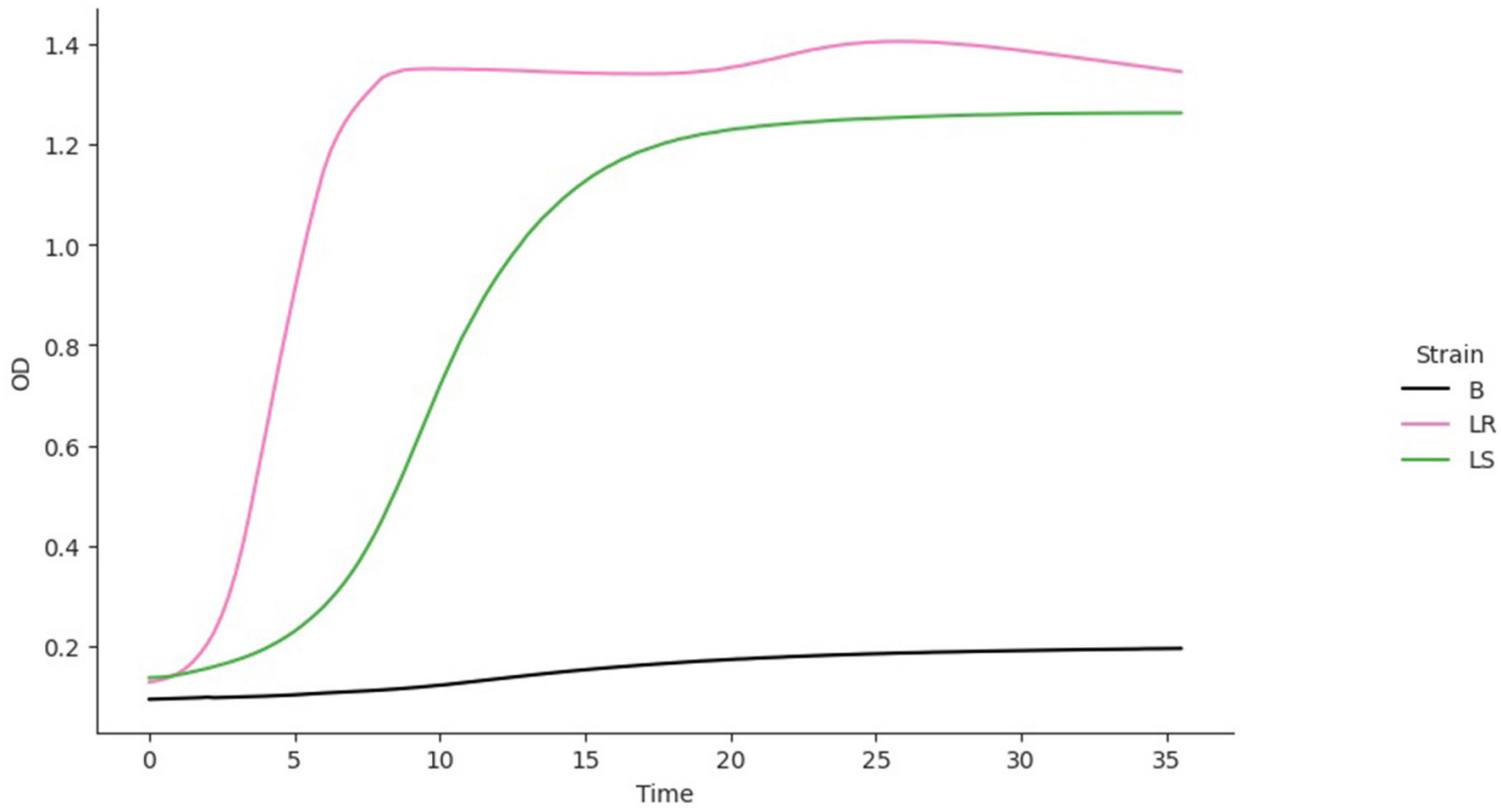
The simulation of competition models from the best model fits of the individual isolates. The estimated growth parameters (*N*_0_*, r, K, v, q*_0_) and *c*_i_ = 0 were used for the prediction. B, Blank; LR, *L. reuteri* ZJ625, *L. salivarius* ZJ625.

The fitting of growth models estimates the growth parameters of the strains and reveals the best-fit models. The data also revealed the Baranyi–Roberts model to be the best fit for *L. salivarius* ZJ614, while *L. reuteri* ZJ625 fits best with the LogisticLag2 model (Figures 3, 4). Also, a summary of the estimated growth parameters showing the minimal specific doubling time, maximum specific growth rate, and lag phase duration are presented in Table 4. The growth parameters deducted were used to simulate the competition behaviors of the strains in a mixed culture (Figure 5).

**Table 4:** Estimated growth parameters for the best models fit of the isolates.

**Strain parameter**	***L. salivarius*** **ZJ614**	***L. reuteri*** **ZJ625**
Initial density (N_0_)	0.15	0.13
Maximum density (OD_600_)	1.26	1.37
Max specific growth rate (hours^−1^)	0.26	0.59
Minimum doubling time (hours)	2.74	1.17
Lag phase duration (hours)	5.51	2.27
Best model fit	Baranyi–Roberts	LogisticLag2

## Discussion

This study determines the nutritional requirements and growth behaviors of *L. salivarius* ZJ614 and *L. reuteri* ZJ625 in CDM and MDM. The importance of chemically defined media for physiological and metabolic studies has been established for several LAB species, including *L. delbrueckii* (Chervaux et al., 2000; Hébert et al., 2004a), *L. helviticus* (Hebert et al., 2000), *Lactococci, Enterococci, Streptococci species* (Zhang et al., 2009), *Lactococcus lactis* (Aller et al., 2014), and other non-LAB members, such as *Bifidobacterium bifidum* (Ferrario et al., 2015). Chemically defined media are useful for high biomass propagation of *Lactobacilli* and are suitable for analytical studies such as proteomics and metabolomics (Zotta et al., 2017). In this study, we modified the validated chemically defined media previously reported to support the growth of different LAB (Ricciardi et al., 2015) to suit *L. salivarius* ZJ614 and *L. reuteri* ZJ625. LAB are auxotrophic for several amino acids required for their growth and may depend on transporters to obtain essential organic substances from their surroundings (Hernandez-Valdes et al., 2020). Previous studies had shown the determination of the auxotrophies of other LAB species (*L. plantarum*) through SOE (Teusink et al., 2005; Wegkamp et al., 2010). A single-omission experiment revealed both isolates to be auxotrophic for some amino acids, including methionine, also reported for other LAB, including *L. lactis* (Hernandez-Valdes et al., 2020). In addition, both strains require isoleucine, arginine, glutamine, asparagine, leucine, glutamic acid, threonine, and histidine for their growth, as previously reported (Kim et al., 2012; Liu et al., 2017). However, the two isolates differ in their requirements for amino acids such as alanine, valine, phenylalanine, glycine, and tyrosine, as shown in Figure 1B. Our findings also showed that some amino acids are stimulatory for the growth of the isolates, with their omissions resulting in slower growth as previously reported (Liu et al., 2017). The inability of the two isolates to grow on media without a complete nutrient group agrees with previous reports of the requirements of LAB's growth to vitamins and associated growth factors such as para-aminobenzoic acid (PABA), biotin, riboflavin, thiamine, vitamin B6, and vitamin B12 (Levering et al., 2016). However, the bacterial strains in this study grew well on media devoid of PABA and biotin, indicating the presence of pathways to substitute for those growth factors. Furthermore, LAB require purine and pyrimidine bases as precursors for the nucleic acid synthesis which are mostly stimulatory for growth except for uracil that is essential (Figure 1D).

The measurement of OD for growth curve experiments is easier to take and requires fewer resources than pairwise competition experiments requiring cell counts to be performed (Concepción-Acevedo et al., 2015; Wiser and Lenski, 2015; Ram et al., 2019). Parametric models of microbial population growth employ a sigmoidal growth function with three factors: lag phase, a period of little or slow growth; log phase characterized by maximum proliferation rate during logarithmic growth (a phase of rapid growth); and the stationary phase (asymptomatic carrying capacity phase), resulting from exhaustion of nutrients in the stationary phase (Zwietering et al., 1990; Baranyi and Roberts, 1995; Tonner et al., 2017). The area under the growth curve (AUC) is another important component of the growth curve (Todor et al., 2014). In addition, the calculation of the AUC can integrate different growth phases into a single parameter that correlates with the growth rate and maximum population (Sprouffske and Wagner, 2016). The growth curve analyses revealed *L. reuteri* ZJ625 to have a shorter lag phase, higher maximum growth rate, and lower minimum doubling time than *L. salivarius* ZJ614, thus indicating the potential of *L. reuteri* ZJ625 to outgrow *L. salivarius* ZJ614 in a mixed culture as predicted by the competition model (Figure 5). The data also revealed the Baranyi–Roberts model to be the best fit for *L. salivarius* ZJ614, while *L. reuteri* ZJ625 fitted best with the LogisticLag2 model (Figures 3, 4).

The models having the lowest BIC were chosen as the best fits for the isolates because of the simplicity and flexibility associated with the BIC (Ram et al., 2019). The Baranyi–Roberts model (Baranyi and Roberts, 1994) is suitable for modeling growth comprising different growth phases, including lag, exponential, deceleration, and stationary phases (Hall et al., 2014). The model assumes that the cell population increases as they adapt to the new environments, decrease as nutrient scarcity looms in, and stop growing with resource exhaustion (Williams, 1967; Ram et al., 2019). Similarly, the logistic model provides a good mathematical explanation of different biological population dynamics, including microbial, plant, and animal growths (Omer, 2018). The logistic model assumes that the population growth rate depends on individuals at a particular time. Modeling bacterial growth behavior showed that the lag phase is harder to predict than the specific growth rate, primarily because of the physiological state and, to a lesser degree, the size of the inoculum (Baty et al., 2002; Pla et al., 2015). However, the growth of the isolates in this study was adjusted to 0.5 McFarland's standard before inoculation in CDM to avert the influence of initial inoculum size on the lag phase and growth rates. Therefore, the prolonged lag phase observed for *L. salivarius* ZJ614 compared with *L. reuteri* ZJ625 could be due to the isolates' physiological state in adjusting to the new growth environment. According to Baranyi and Pin (1999), the effect of the inoculum size becomes more vivid when the CFU is lower than 10^2^-10^3^ cells which was not the case in this study (Table 2). Some bacteria may adopt a long lag phase as a survival strategy to wade off the effects of stressors such as antibiotics in their environments; a long lag phase may not substantially affect the overall population (Moreno-Gámez et al., 2020). Moreno-Gámez et al. (2020) further explained that while bacteria may dominate population increase with the shortest lag phase, tolerance to environmental stressors is proffered by bacteria with a longer lag phase. The differential entry into the stationary phase by the isolates was also observed when the isolates were grown in MRS broth, and the same was also observed for growth in the CDM. The growth models analyses further support the findings of Dlamini et al. (2019) on the ability of *L. reuteri* ZJ625 and *L. salivarius* ZJ614 to survive and thrive when administered to pigs in a coculture. In this study, the LogisticLag2, a derivative of the Lotka–Volterra and Baranyi–Roberts models, described best the growth of *L. reuteri* ZJ625 and *L. salivarius* ZJ614, respectively. These findings are in congruence with those of Fujikawa et al. (2014) and Gimenez and Dalgaard (2004), in which the same models fit best the growth of other bacteria in mixed cultures.

This study determined the essential nutritional requirements of *L. reuteri* ZJ625 and *L. salivarius* ZJ614 in CDM and MDM (through SOE). The isolates expressed appreciable growth in the media (MDM) devoid of some minerals, amino acids, and vitamins. The growth kinetics of the isolates in the CDM revealed *L. reuteri* ZJ625 to have a shorter lag phase duration, higher maximum density, lower minimum doubling time, and higher specific growth rate than *L. salivarius* ZJ614. However, *L. salivarius* ZJ614 also grew in the CDM as observed on MRS, thus indicating the CDM's suitability for the growth of the isolates. The analysis of the growth curve showed LogisticLag2, the model with the lowest growth parameters, the best for *L. reuteri* ZJ625. In contrast, the model with the highest number of parameters, the Baranyi–Roberts model, fitted best the growth of *L. salivarius* ZJ614. The CDM and MDM developed in this study support the growths of both *L. reuteri* ZJ614 and *L. salivarius* ZJ614, and the growth curve analysis revealed the behaviors of the isolates in the CDM.

## Data Availability Statement

The original contributions presented in the study are included in the article/Supplementary Material, further inquiries can be directed to the corresponding author/s.

## Author Contributions

OA and MA conceived the work. OA, MA, MO, and IK designed and drafted the proposal for the research. IK executed the wet-lab aspect of the work as part of his PhD research under the supervision of OA, MA, and MO. IK summarized and analyzed the data generated, drafted, and edited the first manuscript. All authors have read and contributed equally to the final manuscript and append their approval for its publication.

## Funding

This work was funded by the South Africa Agricultural Research Council—Animal Production Parliamentary Grant (Cost center: P02000032) and the article processing charges (APC) was paid by the College of Agriculture, Engineering and Sciences, University of KwaZulu-Natal, South Africa.

## Conflict of Interest

The authors declare that the research was conducted in the absence of any commercial or financial relationships that could be construed as a potential conflict of interest.

## Publisher's Note

All claims expressed in this article are solely those of the authors and do not necessarily represent those of their affiliated organizations, or those of the publisher, the editors and the reviewers. Any product that may be evaluated in this article, or claim that may be made by its manufacturer, is not guaranteed or endorsed by the publisher.
